# Ultrasound waves in tumors via needle irradiation for precise medicine

**DOI:** 10.1038/s41598-022-10407-5

**Published:** 2022-04-20

**Authors:** Antonello Cutolo, Angelo Rosario Carotenuto, Maria Alessandra Cutolo, Arsenio Cutolo, Martino Giaquinto, Stefania Palumbo, Andrea Cusano, Massimiliano Fraldi

**Affiliations:** 1 Department of Electrical Engineering and Information Technology, University of Napoli ″Federico II″, Napoli, Italy; 2 Department of Structures for Engineering and Architecture, University of Napoli ″Federico II″, Napoli, Italy; 3 Optoelectronics Group, Department of Engineering, University of Sannio, Benevento, Italy

**Keywords:** Biomedical engineering, Electrical and electronic engineering, Mechanical engineering

## Abstract

Grounded in the interdisciplinary crosstalk among physics and biological sciences, precision medicine-based diagnosis and treatment strategies have recently gained great attention for the actual applicability of new engineered approaches in many medical fields, particularly in oncology. Within this framework, the use of ultrasounds employed to attack cancer cells in tumors to induce possible mechanical damage at different scales has received growing attention from scholars and scientists worldwide. With these considerations in mind, on the basis of ad hoc elastodynamic solutions and numerical simulations, we propose a pilot study for in silico modeling of the propagation of ultrasound waves inside tissues, with the aim of selecting proper frequencies and powers to be irradiated locally through a new teragnostic platform based on Lab-on-Fiber technology, baptized as a *hospital in the needle* and already the object of a patent. It is felt that the outcomes and the related biophysical insights gained from the analyses could pave the way for envisaging new integrated diagnostic and therapeutic approaches that might play a central role in future applications of precise medicine, starting from the growing synergy among physics, engineering and biology.

## Introduction

The need to reduce collateral effects for patients has progressively occupied a prominent position in the optimization of a vast category of clinical applications. To this aim, precision medicine^1–5^ has become a strategic target to reduce drug doses delivered to the patient by essentially following two main approaches. The first one is based on treatments designed according to the genomic characteristics of the patient. The second one is aimed to avoid a systemic drug delivery procedure by trying to release minor drug quantities but with higher precision by also exploiting localized treatments, which is becoming the gold standard in oncology. The ultimate goal is to cancel or at least minimize the negative side effects of many therapeutic approaches, such as systemic delivery of either chemotherapeutic or radionuclide drugs. Even radiotherapy can involve high intrinsic risks for healthy tissues, depending on the type of cancer, its location, the radiation dose, and other factors. In the treatment of glioblastoma^6–9^, surgery succeeds in removing the main cancer, but even in the absence of metastases, many small cancer infiltrations can be present. If they are not completely removed, new cancer masses can be regenerated in a relatively short period of time. In this case, the aforementioned precision medicine strategies are very hard to apply because these infiltrations are difficult to detect and are distributed over a fairly large region. These obstacles hinder the possibility of obtaining a definitive outcome that prevents any recurrence through precision medicine so that a systemic drug delivery approach is however preferred in several circumstances, although the employed drugs could have a dramatically high toxicity level. To overcome this problem, an ideal therapy would imply the adoption of minimally invasive strategies capable of selectively attacking cancer cells by preserving the healthy tissue. On this line of argument, a possible solution seems to be offered by the use of ultrasonic vibrations, which have been demonstrated to have a different effect on cancer and healthy cells, both in single-cell systems and in cell heterotypic clusters at the mesoscale^10–12^.

From a mechanical point of view, healthy and cancer cells in fact exhibit distinct natural resonance frequencies. This property is associated with a tumorigenic alteration of the mechanical properties of cancer cells’ cytoskeleton structures^12,13^, according to which tumor cells are, on average, more deformable than normal cells. Consequently, through the optimal choice of the ultrasound frequency of stimulation, oscillations induced in a selected area might cause damage to living cancer structures by minimizing the effects in the healthy host surroundings. These not yet completely understood effects could include the disruption of some cell structural components because of the high frequency vibrations due to ultrasounds (in principle, very similar to lithotripsy^14^) as well as cell damage induced by mechanical fatigue-like phenomena, which may in turn alter the cell program and mechanobiology. Despite this theoretical solution looks very suitable, unfortunately, it could not be adopted in cases in which anechoic biological structures impede the direct administration of ultrasounds, such as in intraskull applications, because of the presence of bone, as well as for some breast tumor masses placed in positions where the attenuation of adipose tissue could limit the potential therapeutic effectiveness. To overcome these problems, a localized application of ultrasound is needed by means of ad hoc conceived probes able to reach the irradiation site as minimally invasive as possible. With this in mind, we have thought to take advantage of an idea related to the possibility of creating an innovative technological platform, called “the hospital in the needle”^15^. The ‘hospital in the needle’ concept envisions the development of a minimally invasive medical tool for diagnostics and therapy applications, based on the integration of different functionalities in a single medical needle. As more extensively discussed in “The hospital in the needle” section, such a compact device mainly relies onto the advantages offered by optical fiber based probes^16–21^, which, thanks to their intrinsic characteristics, are suitable to be inserted into the lumen of a standard medical needle^20,22^. By exploiting the flexibility offered by the Lab-on-Fiber (LOF) technology^23^, optical fibers are in fact emerging as unique platforms for the realization of miniaturized and plug-and-play devices for both diagnostics and therapy, including liquid and tissues biopsy based on the detection of relevant bio-molecules^24,25^, light controlled localized drug delivery^26,27^, high precision localized ultrasound based imaging^28^, thermal treatments^29,30^ and recognition of cancer tissues based on optical spectroscopy^31^. In this framework, by taking advantage from the localized approach that is at the basis of the “hospital in the needle” device, we studied the possibility of driving ultrasound waves inside the area of interest by exploiting their propagation through the needle, thus optimizing the localized stimulation of resident biological structures. In this way, it is possible to directly apply low-intensity therapeutic ultrasounds with minimal invasiveness in risky regions to sonicate cells as well as small solid masses in soft tissues, such as in the aforementioned case of intraskull operations, in which a small hole inside the skull is required to let the needle insertion. Motivated by recent theoretical results and experimental findings reporting that ultrasounds might have the potential to arrest or delay the development of some types of cancer^32–34^, the proposed approach might turn instrumental to resolve, at least in principle, the critical trade-off between invasiveness and the impact of the cure. Under these considerations, in the present work, we explored the possibility of using the hospital in the needle device for a minimal invasive ultrasound-based cancer therapy. More precisely, in “Scattering analysis of spherical tumor masses for estimating growth-dependent ultrasound frequencies” section, we make use of well-established methods of elastodynamics and theory of acoustic scattering for predicting the resonance frequencies of spheroidal solid tumors grown in elastic environments to drive the oscillation of the actuator, by exploiting the stiffness discrepancies developing between tumor and host tissues as a result of growth-induced material remodelling. After describing in “The hospital in the needle” section the system that we call “hospital in the needle”, we analyse in “A needle configuration for ultrasound guiding” section the propagation of ultrasonic waves at the predicted frequencies through a medical needle and their irradiation in the surrounding medium with the help of a numerical model in order to examine the main geometrical parameters (actually the inner diameter, the length and the sharpness of the needle), which influence the transmission of acoustic power from the instrument. In the light of the need to conceive new engineered strategies for precise medicine, it is felt that the proposed study could help to design a novel tool for cancer treatment based on the use of ultrasounds administered through an integrated teragnostic platform which combines sonication with other solutions, such as targeted drug delivery and real-time diagnostics, within a single needle.

## Scattering analysis of spherical tumor masses for estimating growth-dependent ultrasound frequencies

### Scattering waves in tumor-host systems

The effectiveness of mechanics-based strategies that provide the use of ultrasound (US) stimulation for treating localized solid tumors has been the object of several papers, which focus on the effects of low-intensity US vibrations on single-cell systems from both a theoretical and experimental point of view^10–12,32–36^. By taking advantage of a viscoelastic model, some researchers have analytically demonstrated that tumor and healthy cells exhibit a different in-frequency response, characterized by distinct resonance-like peaks in the US range^10–12^. This result suggests that, in principle, tumor cells can be selectively attacked by means of mechanical stimulation by preserving the host surroundings. Such behavior was a direct consequence of key evidences according to which tumor cells are, in most cases, more compliant than their healthy counterparts, probably to enhance their proliferation and migration capabilities^37–40^. On the basis of the results obtained from single-cell models, say at the microscale, selectivity of cancer cells was also demonstrated at the mesoscale by numerically studying the harmonic response of heterotypic cell aggregates^12^. Multicellular agglomerates of few hundreds of microns in size were hierarchically built up by providing the presence of cancer and healthy cells at different percentages. At the intermediate scale of these aggregates, some microscopic features of interest were preserved by directly implementing the leading structural elements characterizing the mechanical behavior of single-cells. In particular, each cell incorporated tensegrity-based architectures to simulate the response of the differently prestressed cytoskeleton structures, influencing their overall stiffness ^12,13^. The promising outcomes provided by both theoretical predictions and in vitro experiments from the above mentioned literature works suggest us to investigate the sensitivity of tumor masses to Low Intensity Therapeutic Ultrasounds (LITUS), the evaluation of frequencies at which the tumor mass should be irradiated being crucial for orienting the application of LITUS in situ through engineered solutions for precise medicine.

However, at the tissue level, the sub-macroscopic description of the single constituents is inevitably lost and tumor tissue properties can be traced back by following the mass growth and stress-driven remodeling processes by means of a continuum approach, so taking into account the effect of growth-induced modifications of tissue elasticity at the macroscale occurring during tumor development^41,42^. In fact, unlike single-cell and aggregate systems, solid tumor masses grow in soft tissues by progressively accumulating abnormal residual stresses that alter the native mechanical properties by increasing the overall intratumoral stiffness, tumor stiffening often becoming a determinant for tumor detection.

In light of these considerations, we here analyze the sonodynamic response of tumor spheroids modeled as elastic spherical inclusions grown within a normal tissue environment. More precisely, tumor stage-dependent elastic properties are assigned by starting from theoretical and experimental outcomes obtained by some of the present authors in a previous work^41^. Therein, by implementing nonlinear mechanical models coupled with interspecific dynamics^41,43,44^, the evolution of solid tumor spheroids growing in vivo within a heterogeneous environment were investigated, so predicting the development of tumor masses and related intratumoral stresses. As above mentioned, growth (as inelastic pre-stretch) and residual stresses induce the progressive remodeling of the tumor material properties, consequently changing also its acoustic response. Importantly, in Ref.^41^ the co-evolution of growth and solid stresses in tumors was proved by carrying out an experimental campaign on animal models. In particular, the stiffness of breast tumor masses excised at different stages was compared with the one obtained by replicating analogous conditions in silico on finite element models of spheroids with the same dimensions and including the predicted residual stress fields, so confirming the effectiveness of the modelling approach. In this work, the previously obtained theoretical and experimental outcomes are exploited to orient a novel engineered treatment strategy. More specifically, the predicted sizes with the corresponding evolved elastic properties are here calculated and hence used to estimate the frequency ranges at which tumor masses embedded in the host environment are more sensitive. To this purpose, we thus study the dynamic behavior of tumor masses, taken at different stages, in response to US stimulations according to well-established scattering principles, by considering acoustic indicators with the scope of highlighting possible resonance phenomena of the spheroid on the basis of the growth-dependent stiffness discrepancies between tumor and host tissues.

**Figure 1:**
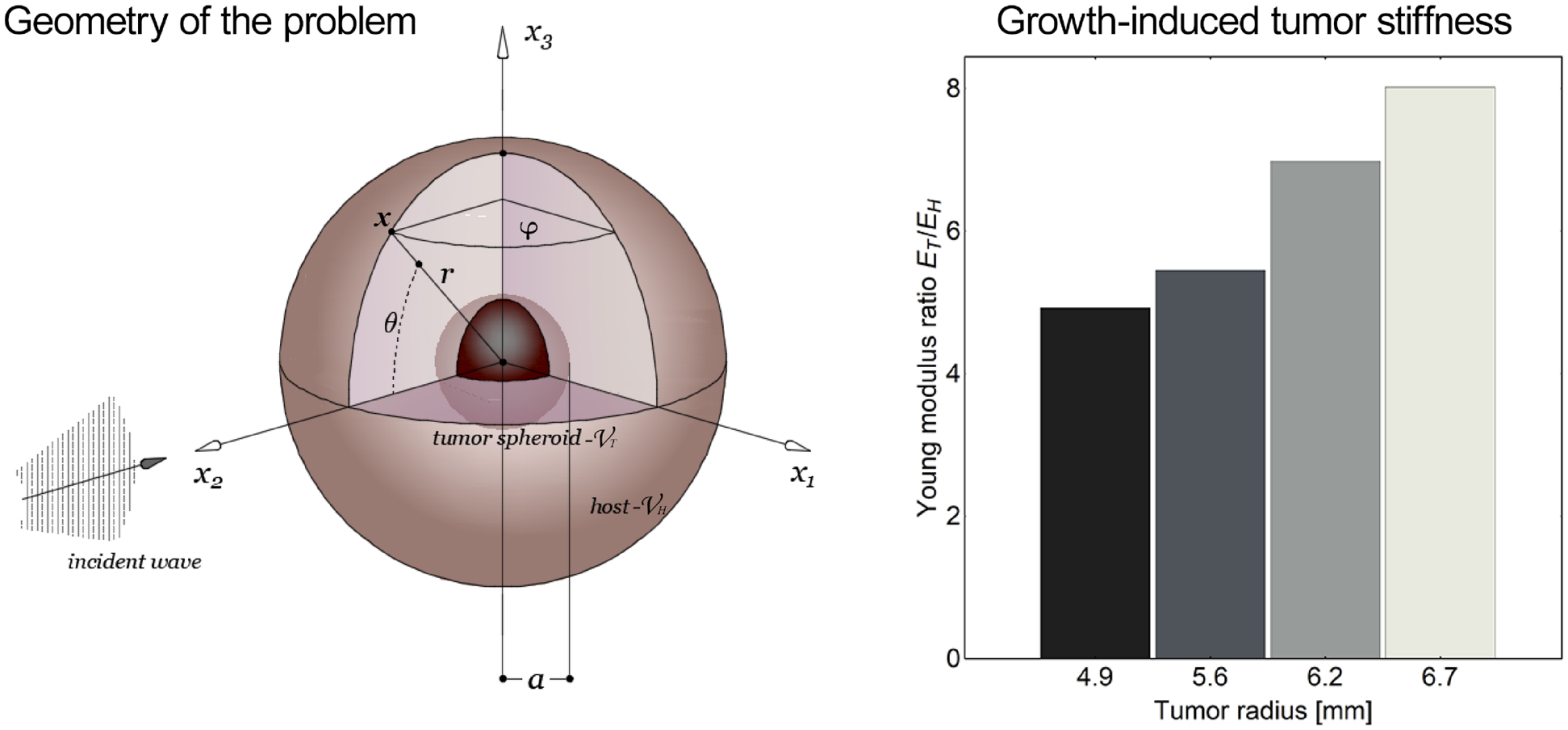
(Left) Geometry of the growing spherical tumor immersed in the healthy surroundings, in which the incident field is propagating and (right) corresponding evolution of the tumor-to-host stiffness ratio as a function of tumor radius, the reported data (adapted from Carotenuto et al.^41^) being obtained from ex vivo compression tests on breast solid tumors obtained from inoculated MDA-MB-231 cells.

Therefore, according to the experimental evidences showing how a vast class of malignant formations grow in situ with a spheroidal shape^41^, the tumor mass was modeled as an elastic sphere of radius $a$ into a surrounding elastic host medium. By making reference to Fig. 1, adopting spherical coordinates $\{ r,\theta ,\varphi \}$ (where $\theta$ and $\varphi$ respectively denote the anomaly and the azimuthal angles), the tumor domain occupies a region ${\mathcal{V}}_{T} = \{ (r,\theta ,\varphi ):r \le a\}$ embedded in the healthy space, identified by the unbounded region ${\mathcal{V}}_{H} = \{ (r,\theta ,\varphi ):r > a\}$. Referring to Supplementary Information (SI) for the complete description of the mathematical model based on well-established elastodynamics framework reported in many literature works^45–48^, we here consider a problem characterized by axially symmetric vibration modes. This assumption implies that all the variables inside the tumor and healthy regions are independent of the azimuthal coordinate $\varphi$ and that no distortions occur along this direction. Consequently, the displacement and stress fields are derivable from the knowledge, in each domain, of two scalar potentials $\phi = \hat{\phi }\left( {r,\theta } \right)e^{{ - i\omega {\kern 1pt} t}}$ and $\chi = \hat{\chi }\left( {r,\theta } \right)e^{{ - i\omega {\kern 1pt} t}}$, which are respectively connected to the longitudinal and shear waves, the anomaly $\theta$ coinciding with the angle between the incident wave direction and the position vector ${\mathbf{x}}$ at each time *t* (as in Fig. 1) and $\omega = 2\pi f$ representing the angular frequency. In particular, the incident field is modelled as a plane wave $\phi_{H}^{(in)}$(also introduced in the SI, in Eq. (A.9)) propagating into the host volume according to the expression

1 $$\phi_{H}^{(in)} = \phi_{0} {\mkern 1mu} e^{{i{\kern 1pt} \left( {r{\kern 1pt} \cos \theta - \omega t} \right)}} ,$$

where $\phi_{0}$ is an amplitude parameter. It is standard argument to perform a spherical expansion of the incident plane wave (1) by using spherical wave functions:

2 $$\phi_{H}^{(in)} = \phi_{0} {\mkern 1mu} e^{{ - i\omega {\kern 1pt} t}} {\mkern 1mu} \sum\limits_{n = 0}^{\infty } {\mkern 1mu} \left( {2n + 1} \right)i^{n} {\mkern 1mu} j_{n} \left( {k_{H1} {\mkern 1mu} r} \right){\mkern 1mu} P_{n} \left( {\cos \theta } \right),$$

in which $j_{n}$ is the spherical Bessel function of the first kind of order $n$ and $P_{n}$ are Legendre polynomials. A part of the incident wave investing the spheroid is scattered into the host medium and overlaps with the incident field, while another part is scattered within the spheroid contributing to its vibration. To this aim, the harmonic solutions of the wave equations $\nabla^{2} \hat{\phi } + k_{1}^{2} {\mkern 1mu} \hat{\phi } = 0\,$ and $\nabla^{2} {\mkern 1mu} \hat{\chi } + k_{2}^{2} \hat{\chi } = 0$ provided, for example, by Eringen^45^ (see also SI) can be particularized to the tumor and healthy regions. Specifically, the scattered dilatational and isochoric waves generated in the host medium $H$ admit the respective potentials:

3 $$\phi_{H}^{(s)} = \phi_{0} {\mkern 1mu} e^{{ - i\omega {\kern 1pt} t}} {\mkern 1mu} \sum\limits_{n = 0}^{\infty } {\mkern 1mu} \left( {2n + 1} \right)i^{n} {\mkern 1mu} \alpha_{n} {\mkern 1mu} h_{n}^{(1)} \left( {k_{H1} {\mkern 1mu} r} \right){\mkern 1mu} P_{n} \left( {\cos \theta } \right),$$

4 $$\chi_{H}^{(s)} = \phi_{0} {\mkern 1mu} e^{{ - i\omega {\kern 1pt} t}} {\mkern 1mu} \sum\limits_{n = 0}^{\infty } {\mkern 1mu} \left( {2n + 1} \right)i^{n} {\mkern 1mu} \beta_{n} {\mkern 1mu} h_{n}^{(1)} \left( {k_{H2} {\mkern 1mu} r} \right){\mkern 1mu} P_{n} \left( {\cos \theta } \right),$$

where the spherical Hankel functions of the first kind $h_{n}^{(1)}$ are adopted to consider outgoing scattered waves, while $\alpha_{n}$ and $\beta_{n}$ are unknown coefficients. In Eqs. (2)–(4), the terms $k_{H1}$ and $k_{H2}$ represent the wavenumbers of the dilatational and shear waves in the host region, respectively (see SI). Inside the tumor, compressional and shear fields are instead given by

5 $$\phi_{T}^{(s)} = \phi_{0} {\mkern 1mu} e^{{ - i\omega {\kern 1pt} t}} {\mkern 1mu} \sum\limits_{n = 0}^{\infty } {\mkern 1mu} \left( {2n + 1} \right)i^{n} {\mkern 1mu} \gamma_{n} {\mkern 1mu} j_{n} \left( {k_{T1} {\mkern 1mu} r} \right){\mkern 1mu} P_{n} \left( {\cos \theta } \right),$$

6 $$\chi_{T}^{(s)} = \phi_{0} {\mkern 1mu} e^{{ - i\omega {\kern 1pt} t}} {\mkern 1mu} \sum\limits_{n = 0}^{\infty } {\mkern 1mu} \left( {2n + 1} \right)i^{n} {\mkern 1mu} \eta_{n} {\mkern 1mu} j_{n} \left( {k_{T2} {\mkern 1mu} r} \right){\mkern 1mu} P_{n} \left( {\cos \theta } \right),$$

where $k_{T1}$ and $k_{T2}$ indicate the longitudinal and transverse wavenumbers in the tumor domain, the unknown coefficients being indicated with $\gamma_{n} {\mkern 1mu}$ and $\eta_{n} {\mkern 1mu}$. From these results, the nonzero radial and circumferential displacement components specialized to the healthy region in the problem at hand—say $u_{Hr}$ and $u_{H\theta }$ ($u_{H\varphi }$ vanishing for symmetry assumptions)—can be obtained through the relations $u_{Hr} = \partial_{r} \left( {\phi + \partial_{r} (r\chi )} \right) + k_{H2}^{2} {\mkern 1mu} r\chi$ and $u_{H\theta } = r^{ - 1} \partial_{\theta } \left( {\phi + \partial_{r} (r\chi )} \right)$ by posing $\phi = \phi_{H}^{(in)} + \phi_{H}^{(s)}$ and $\chi = \chi_{H}^{(s)}$(see SI for detailed mathematical derivations). Analogously, the substitutions $\phi = \phi_{T}^{(s)}$ and $\chi = \chi_{T}^{(s)}$ return the radial and circumferential displacements arising in the tumor domain, i.e. $u_{Tr} = \partial_{r} \left( {\phi + \partial_{r} (r\chi )} \right) + k_{T2}^{2} {\mkern 1mu} r\chi$ and $u_{T\theta } = r^{ - 1} \partial_{\theta } \left( {\phi + \partial_{r} (r\chi )} \right)$.

Assuming linearly elastic and isotropic materials, the nonzero stress components in the healthy and tumor domains—i.e. $\sigma_{Hpq}$ and $\sigma_{Tpq}$—obey the generalized Hooke’s law, by accounting for the presence of distinct Lamé moduli characterizing host and tumor elasticity, respectively denoted by $\{ \mu_{H} ,\,\lambda_{H} \}$ and $\{ \mu_{T} ,\,\lambda_{T} \}$ (the full expression of stress components being reported in SI, see Eq. (A.11)). In particular, growing tumors exhibited evolving tissue elastic constants, according to data from Ref.^41^ and reported in Fig. 1. The displacements and stresses are therefore completely determined in the host and tumor regions up to the sets of unknown constants ${{\varvec{\upxi}}}_{n} = \{ \alpha_{n} ,{\mkern 1mu} \beta_{n} {\mkern 1mu} \gamma_{n} ,\eta_{n} \}$, with theoretically infinite dimensions. To find these coefficient vectors, suitable interfacial and boundary conditions between the tumor and healthy regions are introduced. By assuming perfect bond at the tumor-host interface $r = a$, the continuity of displacements and stresses are required through the following conditions:

7 $$\begin{array}{*{20}l} {\left. {\sigma_{Trr} } \right|_{r = a} = \left. {\sigma_{Hrr} } \right|_{r = a} } \hfill \\ {\left. {u_{Tr} } \right|_{r = a} = \left. {u_{Hr} } \right|_{r = a} } \hfill \\ {\left. {\sigma_{Tr\theta } } \right|_{r = a} = \left. {\sigma_{Hr\theta } } \right|_{r = a} } \hfill \\ {\left. {u_{T\theta } } \right|_{r = a} = \left. {u_{H\theta } } \right|_{r = a} } \hfill \\ \end{array} .$$

System (7) forms a set of equations with infinite solutions. Additionally, each boundary condition will depend on the anomaly $\theta$. To both reduce the boundary value problem to a fully algebraic problem with $N$ sets of closed systems, each of them in the unknowns ${{\varvec{\upxi}}}_{n} = \{ \alpha_{n} ,{\mkern 1mu} \beta_{n} {\mkern 1mu} \gamma_{n} ,\eta_{n} \}_{n = 0,...,N}$ (with $N \to \infty$, theoretically), and to eliminate the dependence of equations on trigonometric terms, the interfacial conditions are written in a weak form by exploiting the orthogonality of the Legendre polynomials. In particular, Eqs. (7)_1,2_ and (7)_3,4_ have been respectively multiplied by $P_{n} \left( {\cos \theta } \right)$ and $P_{n}^{1} \left( {\cos \theta } \right)$ and then integrated between $0$ and $\pi$ with the help of the mathematical identity:

8 $$\int_{0}^{\pi } {P_{n}^{m} } {\mkern 1mu} P_{q}^{k} {\mkern 1mu} \sin \theta {\mkern 1mu} d\theta = \frac{2(n + m)!}{{(2n + 1)(n - m)!}}\delta_{nq} \delta_{mk} .$$

In such a way, the interfacial conditions (7) return square systems of algebraic equations that can be put in matrix form as  ${\mathbb{D}}_{n} (a) \cdot {{\varvec{\upxi}}}_{n} = {\mathbf{q}}_{n} (a)$ and solved through Cramer’s rule to obtain the unknowns ${{\varvec{\upxi}}}_{n}$.

### Acoustic indicators

To evaluate the energy flux scattered by the spheroid and obtain information about its acoustic response from the knowledge of the scattered field propagating in the host medium, the acoustic quantity of interest is the normalized bistatic scattering cross section^49–52^. In particular, the scattering cross section—denoted by $s$—represents the ratio between the acoustic power transported by the scattered signal divided by the rate of energy carried out by the incident wave. In this regard, the form function amplitude $\left| {F_{\infty } \left( \theta \right)} \right|^{2}$ is a generally adopted quantity in the study of the mechanisms of acoustic scattering by objects embedded in fluids or solid sediments. More precisely, the form function amplitude is defined as the differential scattering cross section $ds$ per unit area individuated by the normal to the direction of propagation of the incident wave:

9 $$\left| {F_{\infty } \left( \theta \right)} \right|^{2} = \frac{ds}{{a^{2} d\theta d\varphi }} = \frac{4}{{(k_{H1} a)^{2} }}\left[ {\left| {\sum\limits_{n = 0}^{\infty } {f_{n}^{pp} } \left( \theta \right)} \right|^{2} + \frac{{k_{H1} }}{{k_{H2} }}\left| {\sum\limits_{n = 0}^{\infty } {f_{n}^{ps} } \left( \theta \right)} \right|^{2} } \right],$$

where $f_{n}^{pp}$ and $f_{n}^{ps}$ denote the modal form functions, which refer to the power ratios due to longitudinal and scattered waves with respect to the incident P-wave in the host medium, respectively, given by the expressions:

10 $$f_{n}^{pp} \left( \theta \right) = (2n + 1){\mkern 1mu} \alpha_{n} {\mkern 1mu} P_{n} \left( {\cos \theta } \right)\quad \quad f_{n}^{ps} \left( \theta \right) = (2n + 1){\mkern 1mu} \beta_{n} {\mkern 1mu} \frac{\partial }{\partial \theta }P_{n} \left( {\cos \theta } \right).$$

**Figure 2:**
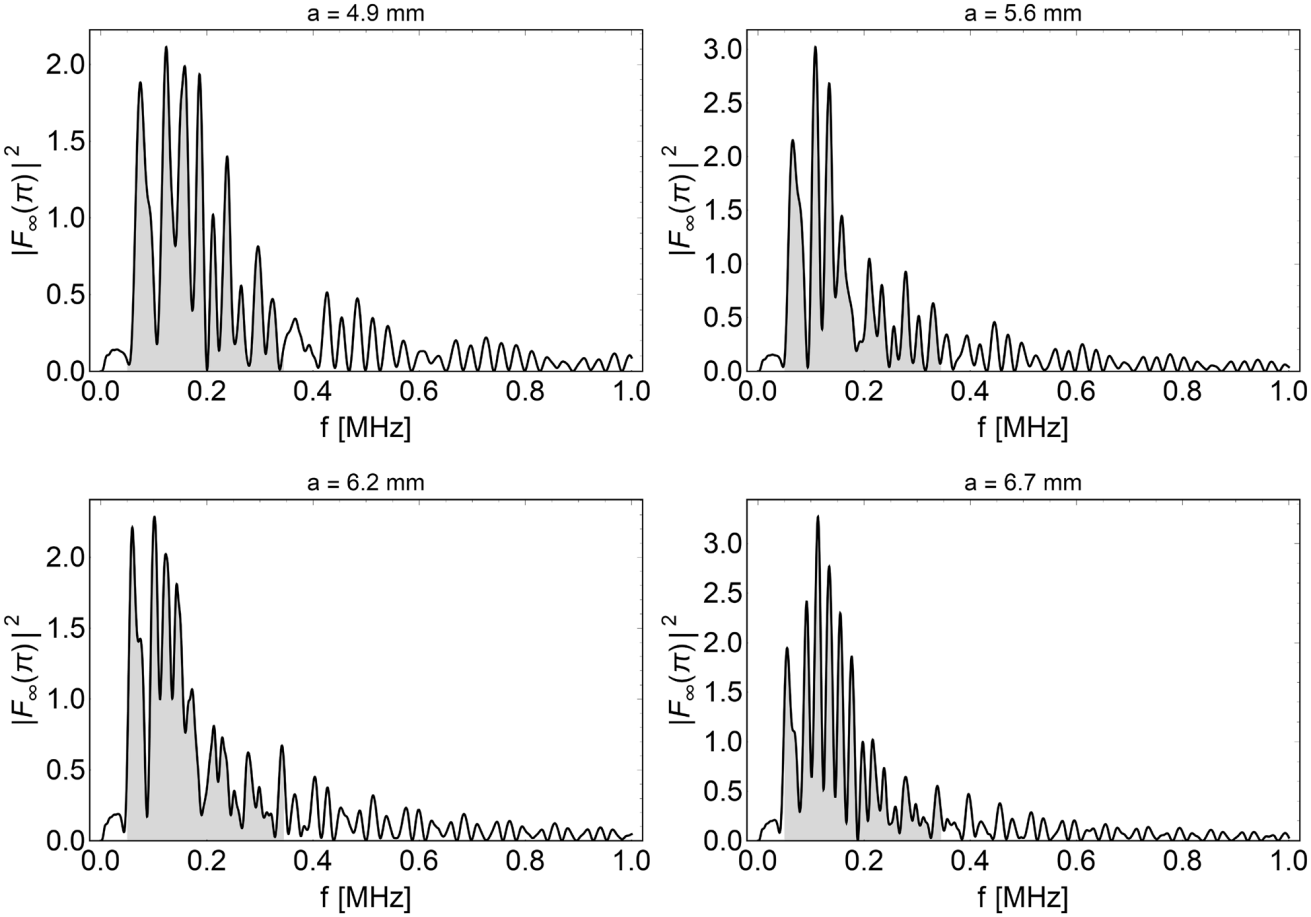
Back scattering form functions obtained from breast tumor moduli reported in Fig. 1 at different sizes, in which the highest scattering band, by considering mode superposition, is highlighted.

**Figure 3:**
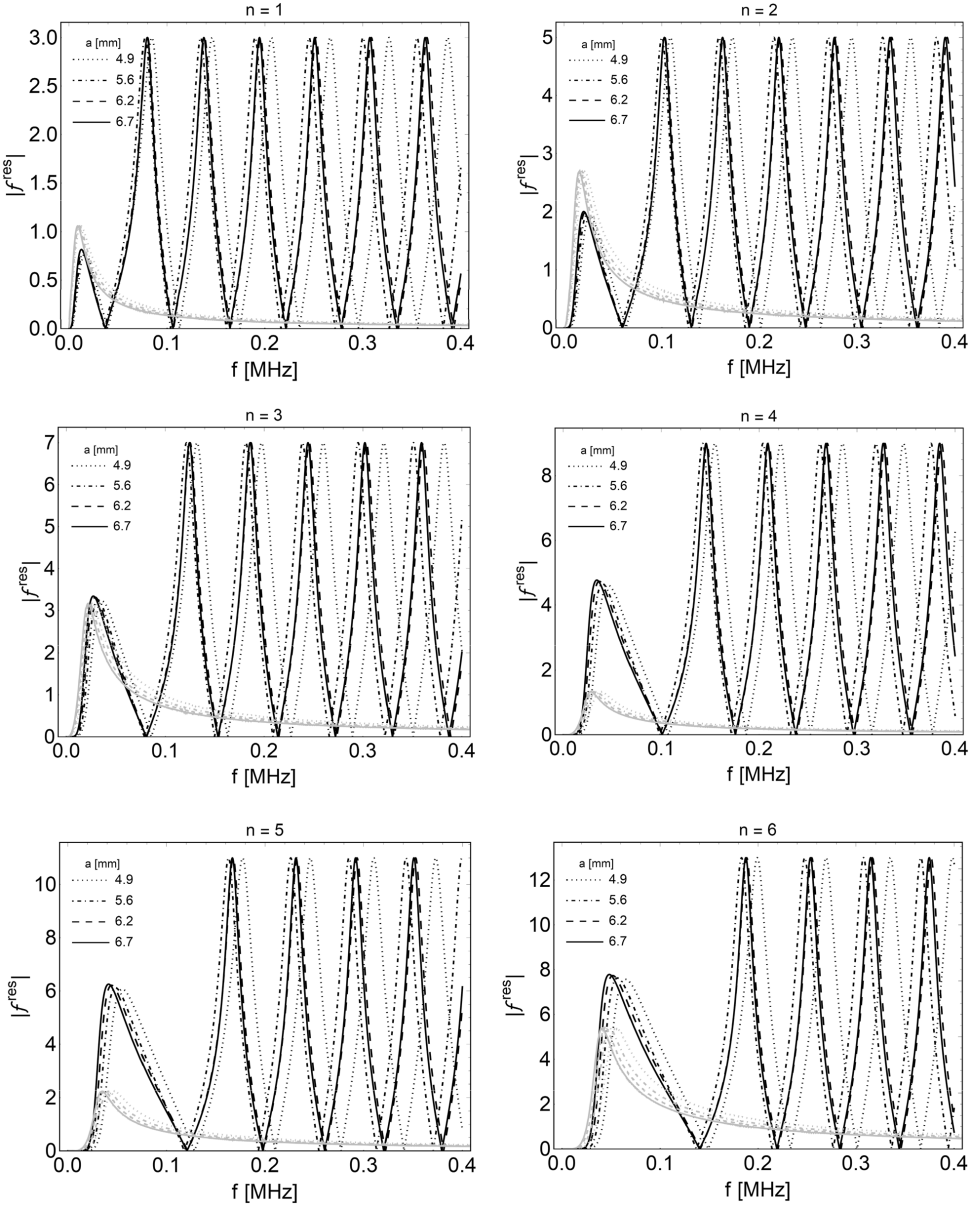
Identification of the resonances for modes $n = 1$ to $n = 6$, calculated at the different tumor sizes in the case of P-wave excitation and P-wave reflection (black curves given by $\left| {f_{n}^{{\left( {res} \right)\,pp}} \left( \pi \right)} \right| = \left| {f_{n}^{pp} \left( \pi \right) - f_{n}^{pp(b)} \left( \pi \right)} \right|$) as well as of P-wave excitation and S-wave reflection (gray curves given by the modal form functions $\left| {f_{n}^{{\left( {res} \right)\,ps}} \left( {\pi /4} \right)} \right| = \left| {f_{n}^{ps} \left( {\pi /4} \right) - f_{n}^{ps(b)} \left( {\pi /4} \right)} \right|$).

The partial waveform functions (10) can be studied separately according to resonance scattering theory (RST)^49–52^, which allows one to separate the elasticity of the target from the total scattered field from the study of the different modes. According to this approach, the modal form functions can be decomposed into the sum of two aliquots, i.e. $f_{n} = f_{n}^{(res)} + f_{n}^{(b)}$, respectively, related to resonance and not resonant background amplitudes. The resonance modal form functions are associated with the response of the target, while the backgrounds are generally related to the scatterer’s shape. With the aim of detecting the first resonance peaks of the target for each mode, the amplitudes of the modal resonance form functions $\left| {f_{n}^{(res)} \left( \theta \right)} \right|$ are computed by assuming a rigid background constituted by an impenetrable sphere in the host elastic material. This hypothesis is motivated by the fact that, generally, both the stiffness and the density can increase during tumor mass growth because of the residual compressive stresses. Consequently, at severe growth levels, the impedance ratio $\rho_{T} c_{1T} /\rho_{H} c_{1H}$ is expected to be greater than 1 for most macroscopic solid tumors developing in soft tissues. As an example, Krouskop et al.^53^ reported a cancer-to-normal ratio of elastic moduli of approximately 4 for prostate tissue, while this value increased to 20 for breast tissue samples. These ratios inevitably produce an alteration of the tissues’ acoustic impedance, as also evincible from elastographic analyses ^54–56^, and could be associated to a local densification of the tissue induced by tumor hyperproliferation. Such differences were also found experimentally from simple compression tests on breast tumor masses grown at different stages ^32^, and the material remodeling was well traced back by predictive interspecific models of nonlinearly growing tumors ^43,44^. The obtained stiffness data are directly correlated to the evolution of the solid tumor Young modulus through the formula $E_{T} = S\left( {1 - \nu ^{2} } \right)/a\sqrt \varepsilon$ (considering nonadhesive contact of an elastic sphere with radius $a$, stiffness $S$ and Poisson ratio $\nu$ between two rigid plates ^57^, as also reported in Fig. 1). In this way, a measure of the tumor and host acoustic impendences at different growth levels can be obtained. In particular, the elastic modulus of breast tumor masses with volumes ranging from approximately 500 to 1250 mm^3^ resulted in an increase from approximately 10 kPa to 16 kPa, compared in Fig. 1 with a normal tissue modulus of 2 kPa, coherently with the interval of 0.25–4 kPa reported in the literature ^58,59^ for breast tissue samples under vanishing precompression. An almost incompressible Poisson ratio is additionally assumed ^41,60^, implying that the density of the tissue does not significantly change during volumetric growth. In particular, an overall mean mass density $\rho = 945\,{\text{kg}}\,{\text{m}}^{ - 3}$ is adopted ^61^. In light of these considerations, rigid background modes can be assumed with the following expressions:

11 $$f_{n}^{pp(b)} \left( \theta \right) = (2n + 1){\mkern 1mu} \delta_{n} {\mkern 1mu} P_{n} \left( {\cos \theta } \right)\quad \quad f_{n}^{ps(b)} \left( \theta \right) = (2n + 1){\mkern 1mu} \upsilon_{n} {\mkern 1mu} \frac{\partial }{\partial \theta }P_{n} \left( {\cos \theta } \right),$$

where the unknown constants $\widehat{{{\varvec{\upxi}}}}_{n} = \{ \delta_{n} ,\upsilon_{n} \}$ can be calculated by considering the continuity displacements (7)_2,4_, i.e., by solving the algebraic system $\widehat{{\mathbb{D}}}_{n} (a) \cdot \widehat{{{\varvec{\upxi}}}}_{n} = \widehat{{\mathbf{q}}}_{n} (a)$ involving the minor $\widehat{{\mathbb{D}}}_{n} (a) = \{ {\mathbb{D}}_{n} (a)\}_{{\{ (1,3),(1,3)\} }}$ as well as the corresponding reduced column vector $\widehat{{\mathbf{q}}}_{n} (a)$. Provided the knowledge of the backgrounds in Eq. (11), the two back-scattered resonance modal form function amplitudes $\left| {f_{n}^{{\left( {res} \right)\,pp}} \left( \theta \right)} \right| = \left| {f_{n}^{pp} \left( \theta \right) - f_{n}^{pp(b)} \left( \theta \right)} \right|$ and $\left| {f_{n}^{{\left( {res} \right)\,ps}} \left( \theta \right)} \right| = \left| {f_{n}^{ps} \left( \theta \right) - f_{n}^{ps(b)} \left( \theta \right)} \right|$ refer to the combination of P-wave excitation with P- and S-wave reflection, respectively. In what follows, the amplitude of the first one has been evaluated at $\theta = \pi$, while the second has been evaluated at $\theta = \pi /4$. The back-scattering form function $\left| {F_{\infty } \left( \theta \right)} \right|^{2}$ amplitude (10) has been evaluated for different tumor growth radii by uploading the different constitutive properties. From Fig. 2, it is highlighted that resonant features for the tumor spheroids up to approximately 15 mm in diameter are mainly concentrated in the band 50–400 kHz, suggesting that low-frequency ultrasounds can be used to induce resonant-type excitation of the mass. Within this band, the RST analysis instead shows single-mode resonance peaks highlighted in Fig. 3 for modes 1 to 6. Herein, both p–p and p–s scatter waves show first resonant-like peaks occurring at extremely low frequencies, which grow from approximately 20 kHz for mode 1 up to approximately 60 kHz for n = 6, while no marked differences are shown in terms of spheroid radius. The p-s resonant functions then attenuate, while combs of p-p resonant peaks with greater amplitude ensure a periodicity of approximately 60 kHz by exhibiting a shift through higher frequencies as the mode number increases. All analyses were performed by means of the computational software Mathematica^®^^62^.

These preliminary analytical results, which make use of far field propagating conditions, let to guide the choice of the particular excitation frequency of the actuator performed in the numerical simulations below to investigate the effects of microvibrational stresses on the mass. The results suggest that the calibration of the optimal frequency could be stage-specific during tumor growth and could be determined by properly predicting tissue remodeling by means of growth models by exploiting their outcomes to build biomechanically driven strategies involved in the treatment of the disease.

## The hospital in the needle

**Figure 4:**
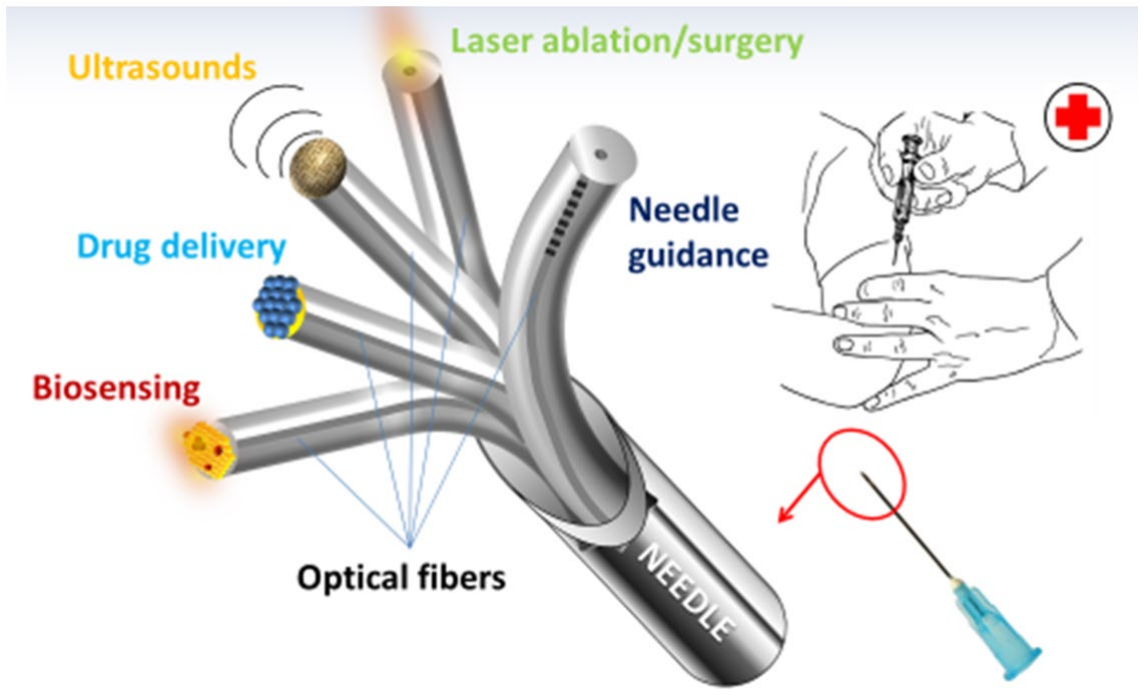
Sketch illustrating the idea of “the hospital in the needle”.

The significant progress of nanotechnologies is leading the scientific community to find new solutions and approaches for the development of miniaturized and minimally invasive medical devices suitable for in-vivo applications. In this context, LOF technology demonstrated an excellent capability of enlarging the functionalities of optical fibers, thus enabling the development of novel minimally invasive optical fiber based devices for life science applications^21,63–65^. The idea of integrating two- and three-dimensional materials with the desired chemical, biological and optical properties on the lateral surface^25^ and/or at the end facet^64^ of an optical fiber with a full spatial control at nanoscale, is leading to a novel class of optical fiber nano-optrodes characterized by a large range of diagnostic and therapeutic functionalities. Interestingly, optical fibers, thanks to their geometrical and mechanical characteristics (small cross section, high aspect ratios, flexibility, low weight) combined with biocompatibility of their material (typically glass or polymers), are well suited to be inserted inside needles and catheters for medical use^20^, paving the way for the new intriguing vision of the “hospital in the needle” (see Fig. 4).

Thanks to the degrees of freedom offered by the LOF Technology, in fact, the optical fibers can be properly functionalized for a specific application, by exploiting the integration of micro- and nano-structures made of different metallic and/or dielectric materials, typically supporting the excitation of resonant modes, which strongly localize the optical field^21^. The light confinement at sub-wavelength scale, often combined with chemical and/or biological treatments^63^, as well as with the integration of responsive materials such as smart polymers^65,66^, allows for an enhanced control of the light-matter interaction, which can be exploited for theranostic purposes. The choice of the type and size of the integrated component/material clearly depends on the physical, biological or chemical parameters to be detected^21,63^.

The integration of LOF probes into a medical needle guided at specific locations of the human body would enable the possibility to perform in vivo local liquid and tissue biopsies, allowing for the delivery of localized therapies at the same time, with reduced side effects and improved efficacy. Potential functionalities involve the detection of different circulating bio-molecules, including cancer bio-markers or micro RiboNucleic Acids (miRNA)^67^, the recognition of cancer tissues through linear and nonlinear optical spectroscopy, such as that based on Surface Enhanced Raman Scattering (SERS)^31^, high resolution optoacoustic imaging^22,28,68^, laser surgery and ablation^69^, as well as light assisted locoregional drug delivery^27^ and the automatic guidance of the needle in human body^20^. It is worth nothing that although the use of optical fibers allows to avoid typical drawback of ‘classic’ approaches based on electronic components, such as the need of electrical connections and the presence of electromagnetic interferences, thus enabling the effective integration of different LOF probes in a single medical needle, particular attention is strongly required to reduce at minimum detrimental effects such as contaminations, optical interference, physical obstructions, which can give rise to cross-talk effects among the different functionalities. However, it is also true that many of the mentioned functionalities are not required to be active simultaneously. This aspect allows to reduce at minimum the interferences, thus limiting the negative effects on the performances of each probe and on the accuracy of the procedures. These considerations allow us to consider the “hospital in the needle” concept more than a simple vision, setting solid basis for the next generation of theragnostic needles for life science.

Concerning the specific application discussed in this work, in the next section we numerically study the capability of a medical needle of guiding ultrasonic waves into human tissues by exploiting their propagation along its axis.

## A needle configuration for ultrasound guiding

**Figure 5:**
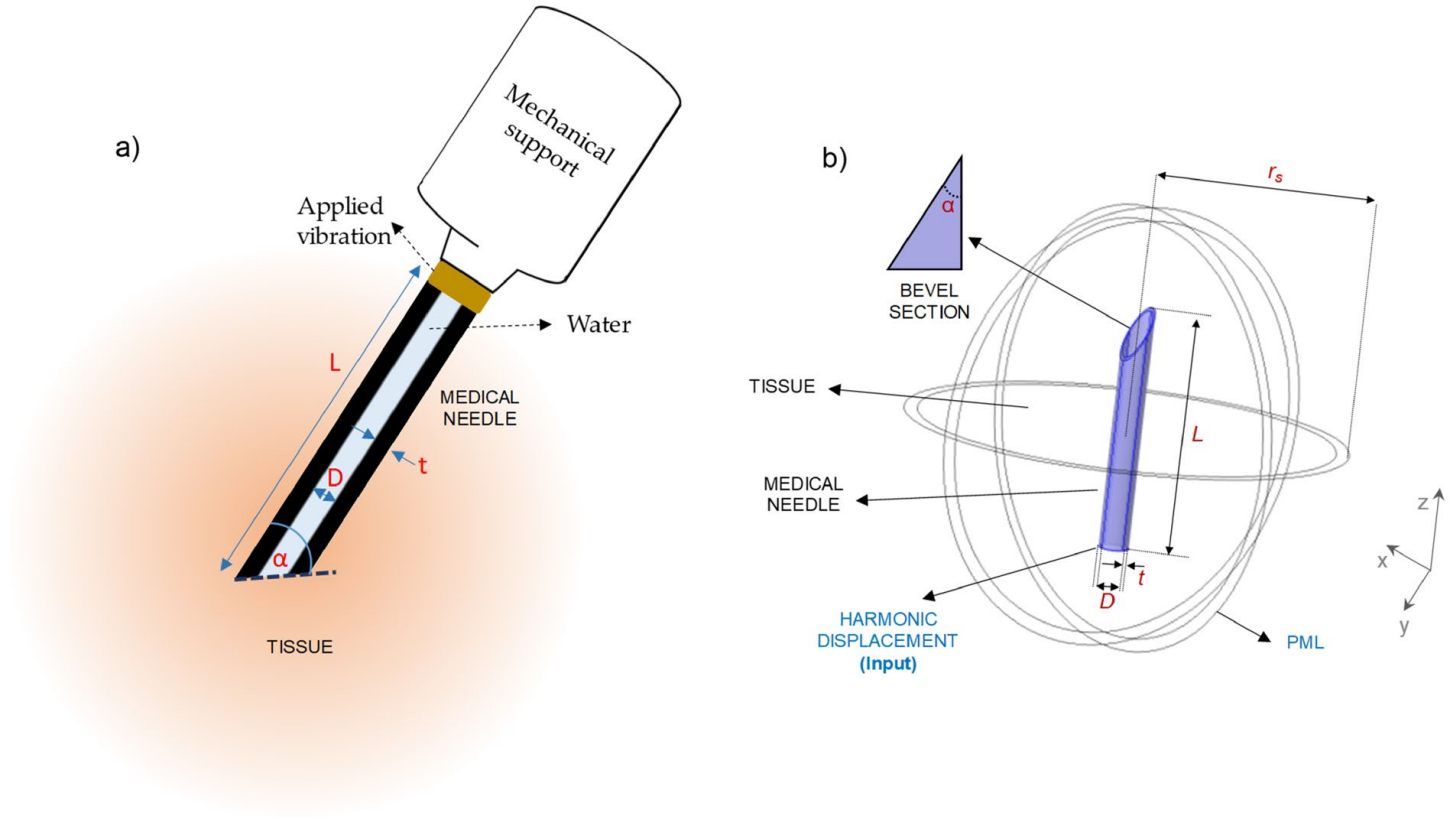
(**a**) Schematic representation of the studied configuration. (**b**) Simulation domains.

The propagation of ultrasonic waves through a medical needle filled with water and inserted in soft tissue (see schematic in Fig. 5a) was simulated by using the commercial software Comsol Multiphysics, based on the finite element method (FEM)^70^, where both the needle and the tissue were modeled as linear elastic media.

With reference to Fig. 5b, the needle is modeled as a hollow cylinder (also referred to as “cannula”) of stainless steel, which is the standard material adopted for medical needles^71^. Specifically it has been modelled with a Young Modulus E = 205 GPa, a Poisson ratio ν = 0.28 and a density ρ = 7850 kg m^−3^^72,73^*.* From a geometrical point of view, the needle is characterized by a length *L,* an inner diameter *D* (also called “lumen”)*,* and a wall thickness *t*. Moreover, the needle tip is considered bevelled of an angle *α* with respect to the longitudinal (*z*) direction. The water domain essentially fits the shape of the needle inner region. In this preliminary analysis, the needle was considered completely immersed in the tissue domain (considered infinitely extended), modeled as a sphere of radius *r*_*s*_, kept constant at 85 mm during all the simulations. More in detail, we terminated the spherical domain with a perfectly matched layer (PML), thus reducing at minimum undesired waves reflection from the ‘fictious’ boundaries. Then, we have chosen the radius *r*_*s*_ in such a way to place the spherical domain boundary far enough from the needle to not influence the computed solution, and small enough to not affect the computational cost of the simulation.

A harmonic longitudinal displacement, with frequency *f* and amplitude *A*, is imposed at the bottom boundary of the needle geometry; this condition constitutes the input stimulus applied to the simulated geometry. On the rest of the needle boundaries (placed in contact with the tissue and the water), a proper condition is imposed by considering that the adopted model involves the coupling between two physics, one pertaining to structural mechanics (for the needle domain) and one to acoustics (for the water and needle domains)^74^*.* More specifically, the small vibrations applied at the needle base cause small stress perturbations; therefore, assuming that the needle behaves as an elastic medium, the displacement vector ***U*** can be evaluated through the elasto-dynamic equilibrium (Navier’s) equation^75^*.* The structural vibration of the needle causes pressure changes in the water inside it (considered stationary in our model), resulting in a propagation of acoustic waves along the longitudinal direction of the needle, essentially ruled by the Helmholtz equation^76^*.* Finally, in the assumption that nonlinear effects in tissues can be neglected and that the amplitude of shear waves is much smaller than that of pressure waves, the Helmholtz equation can also be considered for modeling acoustic wave propagation inside soft tissue. In the wake of this approximation, the tissue was treated as a fluid with a density of 1000 kg/m^3^ and speed of sound of 1540 m/s (neglecting the frequency-dependent attenuation effects)^77^*.* To couple the two phisics, it is necessary to impose the continuity of the normal displacement at the solid/fluid interfaces, the static equilibrium between the pressure and the stress normal to the solid boundaries, while the tangential stresses at the fluid boundaries must be zero^75^*.*

In our analysis, we studied acoustic wave propagation along the needle under steady-state conditions, focusing on the influence that needle geometrical characteristics have on the wave irradiation inside the tissue. More specifically, we studied the influence of the needle inner diameter *D*, the length *L*, and the bevel angle *α*, while the thickness *t* was kept fixed at 500 μm for all the cases of study. This value of *t* is close to the typical standard wall thicknesses adopted for commercial needles^71^.

Without loss of generality, the frequency *f* of the harmonic displacement applied at the needle base was set equal to 100 kHz, with an amplitude *A* of 1 μm. In particular, the frequency was set to 100 kHz in agreement with the analytical estimations reported in “Scattering analysis of spherical tumor masses for estimating growth-dependent ultrasound frequencies” section, where the resonant-like behavior of the tumor masses is found in a frequency range of 50–400 kHz, with the highest scattering amplitudes concentrating at lower frequencies at about 100–200 kHz (see Fig. 2).

The first investigated parameter is the needle inner diameter *D*. For convenience, it was defined as an integer submultiple of the acoustic wavelength in the needle lumen (i.e. in water, *λ*_*W*_ = 1.5 mm). Wave propagation phenomena in devices characterized by a given geometry (such as in waveguides), in fact, typically depends on the characteristic dimension of the involved geometry compared with the wavelength of the propagating wave. Moreover, in this first analysis, to better underline the role of diameter *D* on acoustic wave propagation through the needle, we considered a flat tip, setting the angle *α* = 90°. During this analysis, the needle length *L* was fixed at 70 mm.

**Figure 6:**
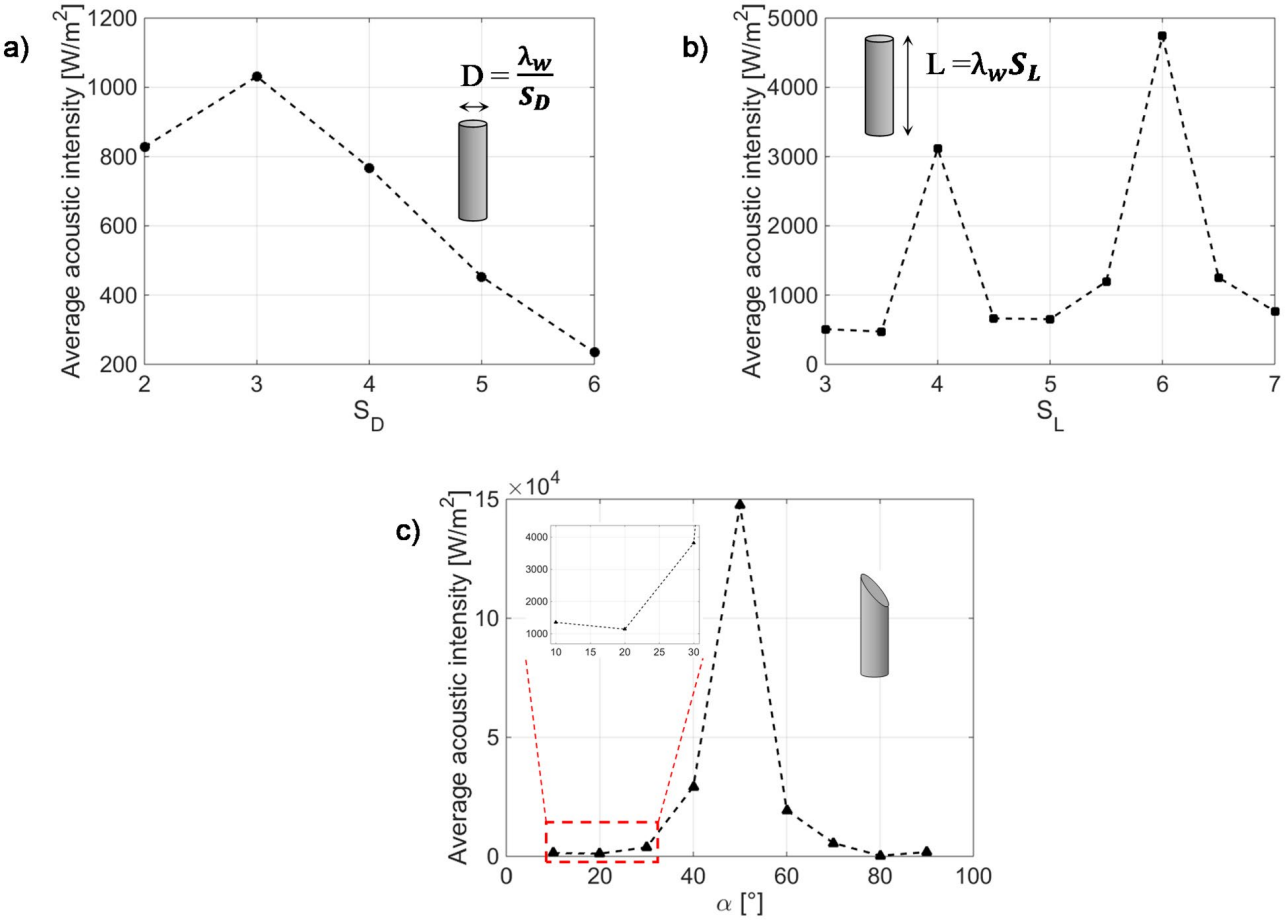
Average acoustic intensity coming out from the needle tip (considered flat) as a function of the needle internal diameter (**a**), length (**b**), and bevel angle α (**c**). The length in (**a,c**) is 90 mm, and the diameter in (**b,c**) is 3 mm.

Figure 6a shows the average acoustic intensity as a function of a dimensionless scale parameter *S*_*D*_, being *D* = *λ*_*W*_*/S*_*D*_*,* evaluated in a sphere of radius 10 mm centered in correspondence of the needle tip. The scale parameter *S*_*D*_ was changed from 2 to 6, i.e., we considered *D* values ranging from 7.5 mm to 2.5 mm (at *f* = 100 kHz). This range also includes standard values adopted for stainless steel medical needles^71^*.* As expected, the needle inner diameter influences the acoustic intensity coming out from the needle, which assumes its maximum (1030 W/m^2^) in correspondence of *D* = *λ*_*W*_/3 (i.e. *D* = 5 mm) and assumes a decreasing trend with the diameter decrease. It is worth considering that the diameter *D* is a geometrical parameter that also influences the invasiveness of the medical device, and for this reason, the choice of the optimum value cannot ignore this crucial aspect. Therefore, although the reduction of *D* is paid with a lower acoustic intensity transmission inside the tissue, for the next studies, a diameter *D* = *λ*_*W*_/5, i.e.,* D* = 3 mm (corresponding to the standard 11G^71^, at *f* = 100 kHz), was considered as a reasonable compromise between the device invasiveness and acoustic intensity transmission (that reaches an average value of about 450 W/m^2^).

The next analyzed parameter is the needle length *L*. Consistently with the previous case of the study, we considered the bevel angle *α* = 90°, and the length was scaled as a multiple of the wavelength in water, i.e. considering *L* = *S*_*L*_* · λ*_*W*_*.* The dimensionless scale parameter *S*_*L*_ was changed from 3 to 7, thus evaluating the average acoustic intensity coming out from the needle tip for lengths ranging from 4.5 to 10.5 mm at the analyzed frequency. This range includes typical values used for commercial needles. The results, shown in Fig. 6b, demonstrate a strong influence of the needle length *L* on the acoustic intensity transmission inside the tissue. Specifically, the optimization of this parameter allows us to improve the transmission of approximately one order of magnitude. In fact, in the analyzed range of lengths, the average acoustic intensity assumes a local maximum of 3116 W/m^2^ at *S*_*L*_ = 4 (i.e., *L* = 60 mm) and another 4743 W/m^2^ corresponding to *S*_*L*_ = 6 (i.e., *L* = 90 mm).

After analyzing the influence of both the diameter and the length of the needle on ultrasound propagation in a cylindrical geometry, we focused on the influence of the bevel on the acoustic intensity transmission inside the tissue. The average acoustic intensity coming out from the fiber tip was evaluated as a function of the angle *α*, changing its value from 10° (sharp tip) to 90° (flat tip). In this case, the considered integration sphere around the needle tip had a radius of 20 mm to ensure that, for all the values of *α,* the needle tip was included in the volume for the average value calculation.

As shown in Fig. 6c, by sharpening the needle tip, i.e., by decreasing *α* starting from 90°, the transmitted acoustic intensity increases, reaching a maximum of approximately 1.5 × 10^5^ W/m^2^ corresponding to *α* = 50°, i.e., 2 order of magnitude higher with respect to the flat condition. By further sharpening the needle tip (i.e., for *α* lower than 50°), the acoustic intensity assumes a decreasing trend, reaching values comparable to those of the flat tip. However, although for our simulations we considered a wide range of bevel angles, it is worth taking into account that tip sharpening is necessary to facilitate needle insertion inside the tissues. Actually, lower bevel angles (approximately 10°) allow for reducing the amount of force required to penetrate tissues^78^*.*

**Figure 7:**
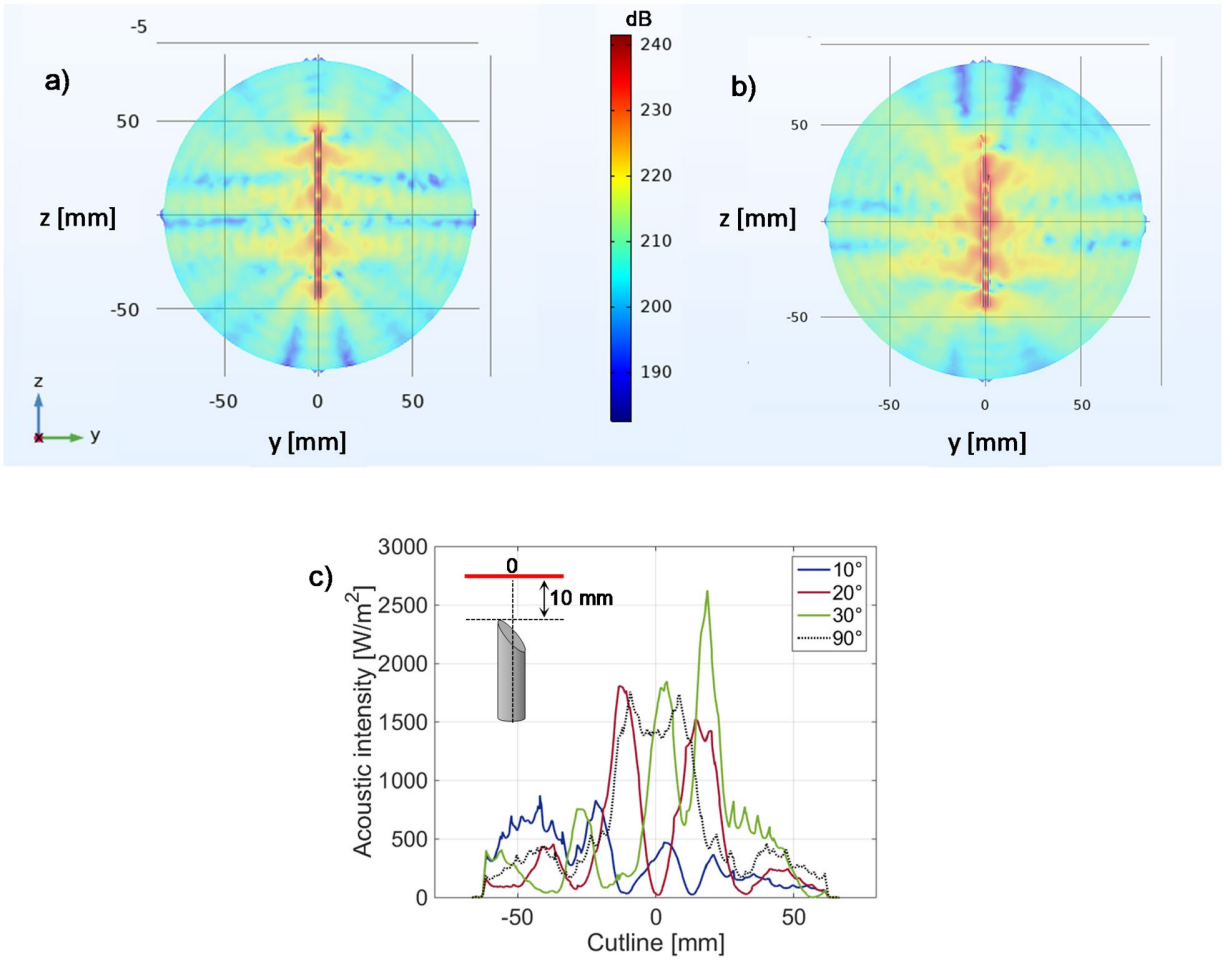
Sound pressure level in the y–z plane of symmetry of the needle in the case of flat tip (**a**) and 10° beveled (**b**). (**c**) Acoustic intensity profiles evaluated along a cutline orthogonal to the needle longitudinal direction, 10 mm distant from the needle tip, and lying in the y–z plane of symmetry. The length L is 70 mm, the diameter D is 3 mm.

In addition to the acoustic intensity value transmitted inside the tissue, the bevel also influences the wave propagation direction, as shown in sound pressure level maps reported in Fig. 7a (for a flat tip) and 3b (for a 10° beveled tip), evaluated in the needle plane of symmetry (y–z, with reference to Fig. 5) parallel to the longitudinal direction. In these two considered extreme cases, the sound pressure level (which is referred to as 1 µPa) is mostly concentrated inside the needle lumen (i.e., in water domain), and is irradiated into the tissue. In more detail, in the case of a flat tip (Fig. 7a), the sound pressure level distribution is perfectly symmetric with respect to the longitudinal direction, and a stationary wave can be distinguished inside the water filling the cannula. This wave is oriented along the longitudinal direction (*z*-axis), with an amplitude that assumes the maximum values inside the water (approximately 240 dB) and decreases along the transverse direction, resulting in an attenuation of approximately 20 dB at a distance of 10 mm from the needle center. The introduction of a sharpen tip (Fig. 7b), as expected, breaks this symmetry, and the antinodes of the stationary waves ‘deflect’ in correspondence of the needle tip. Obviously, this asymmetry affects the intensity coming out from the needle tip, as already discussed (Fig. 6c). To better understand this aspect, the acoustic intensity was evaluated along a cutline orthogonal to the needle longitudinal direction, lying in the needle plane of symmetry, and placed at a distance of 10 mm from the needle tip (results plotted in Fig. 7c). More specifically, the acoustic intensity profiles evaluated at bevel angles of 10°, 20° and 30° (blue, red and green solid lines, respectively) are compared with those pertaining to the flat tip (black dashed curve). The intensity profile pertaining to the needle with a flat tip appears symmetrical with respect to the needle center. In particular, it assumes a value of approximately 1420 W/m^2^ at the center, with an overelongation of approximately 300 W/m^2^ at a distance of ~ 8 mm, and then decreases, reaching a value of approximately 170 W/m^2^ at ~ 30 mm. When the tip is sharpened, the central lobe is split into more lobes of different intensities. More specifically, when α is 30°, three lobes can be clearly distinguished in the profile evaluated at 1 mm distant from the needle tip. The central one is almost located at the needle center and assumes a value of 1850 W/m^2^, while the higher one is at the right side, at a distance of approximately 19 mm from the center, and reaches 2625 W/m^2^. With *α* = 20°, the main lobes become 2, one at − 12 mm of 1785 W/m^2^ and one at 14 mm of 1524 W/m^2^. When the tip becomes sharper and the angle reaches a value of 10°, a maximum of 817 W/m^2^ is reached at approximately − 20 mm, and the other three lobes of a slightly lower intensity are visible along the profile.

Overall, these results demonstrate that a medical needle can be effectively used for transmitting ultrasound waves inside soft tissues at frequencies on the order of 100 kHz. The acoustic intensities irradiated depend on the needle geometrical characteristics and can be optimized (taking into account the constraints imposed by the invasiveness of the final device), reaching values in the range of 1000 W/m^2^ (at a distance of 10 mm) in the case of a displacement of 1 µm applied at the needle base, considered completely inserted in infinitely extended soft tissue. Specifically, the bevel angle strongly influences both the intensity and the propagation direction of the acoustic wave inside the tissue, which results mainly oriented orthogonally to the needle tip cut.

## Conclusions

With the aim of supporting the development of new therapeutic strategies for tumor treatment based on the use of noninvasive medical techniques, the propagation of low-frequency ultrasounds in tumor environments has been analyzed by means of both analytical and computational approaches. In particular, in a first part of the study, ad hoc elastodynamic solutions allowed us to study the scattering of US waves in solid tumor spheroids of known dimension and stiffness to explore the frequency sensitivity of the masses. Frequencies on the order of hundreds of kilohertz were then selected and employed in numerical simulations modeling the localized application of vibrational stresses in the tumor environment through a medical needle actuator by examining the influence of the main design parameters governing the transmission of acoustic power from the instrument toward the surrounding medium. The results suggest that a medical needle can be effectively employed to irradiate ultrasound waves into tissues, with an intensity that is strongly related to the needle geometrical parameters, referred to the operating acoustic wavelength. The intensity irradiated through the tissues, in fact, increases as a function of the needle inner diameter increase, reaching a maximum value when the diameter is threefold higher than the wavelength. The needle length also offers a degree of freedom to optimize the irradiated intensity. The latter results indeed maximized when the needle length is set equal to certain multiples of the operating wavelength (specifically 4 and 6). Interestingly, for the frequency range of interest, the optimized values for the diameter and the length are close to those typically used for standard commercial needles. The bevel angle defining the needle sharpness also has an impact on the irradiated intensity, allowing its maximization for angle values around 50°, also ensuring good performances for angles around 10°, typically used for commercial needles. The outcomes of the simulations will be used to guide the realization and optimization of the teragnostic platform of the hospital in the needle to integrate both diagnostic and therapeutic ultrasounds with other treatment solutions within the instrument for implementing synergistic interventions in the field of precise medicine.

## Supplementary Information

Supplementary Information.
