# Supplementary material for: Ultrasound waves in tumors via needle irradiation for precise medicine
Source: Sci Rep. 2022 Apr 20;12:6513. doi: 10.1038/s41598-022-10407-5 (PMC9021295; doi:10.1038/s41598-022-10407-5)
Supplement: Supplementary file 1 — Supplementary Information. [file 41598_2022_10407_MOESM1_ESM.docx]

**Supplementary information**

**The e*quations of the elastodynamic problem***

The problem is formulated in the firm elastodynamics framework [34] by analyzing the scattering response of the tumor spheroid associated with the individuation of resonant frequencies by exploiting well-established results of the resonance scattering theory [35, 36, 37, 38]. Both tumor and host tissues are assumed to be elastic homogeneous and isotropic media, and in line with literature evidence showing that tumor masses are stiffer than the healthy surroundings, different constitutive properties are assigned. Therefore, the general field equations presented here will be particularized for each material in the following sections. Under isotropy, Navier’s equation of motion involving the vector displacement **u** reads:

$$\left(2\mu+\lambda\right)\nabla\nabla\cdot\mathbf{u}-\mu\text{ }\nabla\times\nabla\times\mathbf{u}+\rho\left(\mathop{\mathbf{u}}\limits^{..} -\mathbf{f}\right)=\mathbf{0}$$

where$\mu$and $\lambda$ are the Lamé moduli of the considered body, that, in the case of viscoelastic materials, can be represented as $\mu=\mu_{e}-j\omega\mu_{v}$ and $\lambda=\lambda_{e}-j\omega\lambda_{v}$. In the following, we will focus on elastic media with density $\rho$ that, in the case of elastically incompressible bodies, does not significantly change during tumor development. The vector is the Nabla differential operator, while the dots denote the time derivative. Considering null body forces $\mathbf{f}$, equation can be rewritten by introducing the Helmholtz representation of the displacements $\mathbf{u}$:

where and $\mathbf{\Psi}\left(\mathbf{x},t\right)$ are the scalar- and vector-valued potentials that are associated, as well known, with the dilatational and equivoluminal motions, respectively. By combining equations with and by imposing the gauge conditions $\nabla\cdot\mathbf{\Psi}=0$, the following two equations are obtained:

in which the irrotational and isochoric wave velocities *c*_1_ and *c*_2_, respectively, associated with the P- and S-waves propagating within the media:

$$c_{1}=\sqrt{\frac{2\mu+\lambda}{\rho}}, c_{2}=\sqrt{\frac{\mu}{\rho}}$$

Considering that the gauge condition reduces the independent components of vector potential $\mathbf{\Psi}\left(\mathbf{x},t\right)$ of one, it can be rewritten as the combination of two further scalar Debye potentials $\psi\left(\mathbf{x},t\right)$ and$\chi\left(\mathbf{x},t\right)$, i.e.,

$$\mathbf{\Psi}=\mathbf{w}\psi\left(\mathbf{x},t\right)+l \nabla\times\left(\mathbf{w}\chi\left(\mathbf{x},t\right)\right)$$

where $\mathbf{w}$is a function of the coordinate pointing toward the direction of propagation, while $l$ is a dimensional parameter. Relation let us decouple equation _2_ into two independent Helmholtz equations of the form:

Equations _1_ and form a set of wave equations that can be rewritten in the Fourier frequency domain as:

in which denotes the Fourier transformation (the hat will be avoided in the following) and the scalars $k_{1}$and $k_{2}$are known as the wavenumbers of the dilatational and shear waves, respectively:

$$k_{1}=\frac{\omega}{c_{1}} \text{and} k_{2}=\frac{\omega}{c_{2}}$$

For the problem at hand, we will consider an incident plane wave with the following expression:

where is a constant, $k_{1}=\left|\mathbf{k}_{1}\right|$ and $k_{2}=\left|\mathbf{k}_{2}\right|$. With a focus on the specific application, the expressions of the potentials, displacement fields and material parameters will be all particularized to either the host surrounding medium or the target tumor spheroid by utilizing subscripts *H* and *T* to denote the related properties, respectively.

According to the experimental observations showing how a vast class of malignant formations grow *in situ* with a spheroidal shape, the tumor mass was modeled as an elastic sphere with radius immersed into a surrounding elastic host medium. In spherical coordinates , it can be shown that in equation , the function $\mathbf{w}=r \mathbf{e}_{r}$and the parameter $l=1$. By further assuming spherical symmetry, the displacement vectors in do not depend on the azimuthal coordinate , and no motion occurs along this direction, i.e.,. To respect this hypothesis, the scalar function $\psi$vanishes. The components of the displacement vector expressed in the spherical frame of reference are thus given by:

Assuming linearly elastic and isotropic materials, the Cauchy stress tensor follows the generalized Hooke’s law $\mathbf{\sigma}=2\mu\text{sym}\left(\nabla\mathbf{u}\right)+\lambda\left(\nabla\cdot\mathbf{u}\right)\mathbf{I}$. In light of the relation , the nonzero stress components result:
